# Periprocedural Myocardial Infarction After Coronary Artery Bypass Grafting: Current Clinical Practices and Future Perspectives

**DOI:** 10.1093/ejcts/ezaf344

**Published:** 2025-10-09

**Authors:** Brian Swinnen, Jules R Olsthoorn, Casper Mihl, Martijn W Smulders, Sandeep K Singh, Thomas van Brakel, Iwan C C van der Horst, Alma M A Mingels, Patrick O Myers, Arnoud W J van ‘tHof, Anton P M Gorgels, Matthias Thielmann, Raffaele De Caterina, Rui J Cerqueira, Nikolaos Bonaros, Wouter Oosterlinck, Steven Jacobs, Roberto Lorusso, Elham Bidar, Joachim E Wildberger, Jos G Maessen, Mario Gaudino, Can Gollmann-Tepeköylü, Samuel Heuts

**Affiliations:** Department of Cardiothoracic Surgery, Maastricht University Medical Centre (MUMC+), Maastricht, The Netherlands; Department of Radiology and Nuclear Medicine, Maastricht University Medical Centre (MUMC+), Maastricht, The Netherlands; Cardiovascular Research Institute Maastricht (CARIM), Maastricht University, Maastricht, The Netherlands; Department of Cardiothoracic Surgery, Catharina Hospital Eindhoven, Eindhoven, The Netherlands; Department of Cardiothoracic Surgery, Isala Heart Centre, Zwolle, The Netherlands; Department of Radiology and Nuclear Medicine, Maastricht University Medical Centre (MUMC+), Maastricht, The Netherlands; Cardiovascular Research Institute Maastricht (CARIM), Maastricht University, Maastricht, The Netherlands; Department of Radiology and Nuclear Medicine, Maastricht University Medical Centre (MUMC+), Maastricht, The Netherlands; Cardiovascular Research Institute Maastricht (CARIM), Maastricht University, Maastricht, The Netherlands; Department of Cardiology, Maastricht University Medical Centre (MUMC+), Maastricht, The Netherlands; Department of Cardiothoracic Surgery, Isala Heart Centre, Zwolle, The Netherlands; Department of Cardiothoracic Surgery, Catharina Hospital Eindhoven, Eindhoven, The Netherlands; Cardiovascular Research Institute Maastricht (CARIM), Maastricht University, Maastricht, The Netherlands; Board of Directors, Albert Schweitzer Hospital, Dordrecht, The Netherlands; Cardiovascular Research Institute Maastricht (CARIM), Maastricht University, Maastricht, The Netherlands; Central Diagnostic Laboratory, Maastricht University Medical Centre (MUMC+), Maastricht, The Netherlands; Department of Cardiac Surgery, CHUV-Centre Hospitalier Universitaire Vaudois, Lausanne, Switzerland; Cardiovascular Research Institute Maastricht (CARIM), Maastricht University, Maastricht, The Netherlands; Department of Cardiology, Maastricht University Medical Centre (MUMC+), Maastricht, The Netherlands; Department of Cardiology, Zuyderland Medical Centre, Heerlen, The Netherlands; Cardiovascular Research Institute Maastricht (CARIM), Maastricht University, Maastricht, The Netherlands; West-German Heart and Vascular Centre, University Duisburg-Essen, Essen, Germany; Cardiology Division, Pisa University Hospital, Pisa, Italy; Department of Surgery and Physiology, Faculty of Medicine, University of Porto, Porto, Portugal; Department of Cardiac Surgery, Innsbruck Medical University, Innsbruck, Austria; Department of Cardiac Surgery, University Hospitals Leuven, Leuven, Belgium; Department of Cardiac Surgery, University Hospitals Leuven, Leuven, Belgium; Department of Cardiothoracic Surgery, Maastricht University Medical Centre (MUMC+), Maastricht, The Netherlands; Cardiovascular Research Institute Maastricht (CARIM), Maastricht University, Maastricht, The Netherlands; Department of Cardiothoracic Surgery, Maastricht University Medical Centre (MUMC+), Maastricht, The Netherlands; Cardiovascular Research Institute Maastricht (CARIM), Maastricht University, Maastricht, The Netherlands; Department of Radiology and Nuclear Medicine, Maastricht University Medical Centre (MUMC+), Maastricht, The Netherlands; Cardiovascular Research Institute Maastricht (CARIM), Maastricht University, Maastricht, The Netherlands; Department of Cardiothoracic Surgery, Maastricht University Medical Centre (MUMC+), Maastricht, The Netherlands; Cardiovascular Research Institute Maastricht (CARIM), Maastricht University, Maastricht, The Netherlands; Department of Cardiothoracic Surgery, Weill Cornell Medicine, New York, NY, United States; Department of Cardiac Surgery, Innsbruck Medical University, Innsbruck, Austria; Department of Cardiothoracic Surgery, Maastricht University Medical Centre (MUMC+), Maastricht, The Netherlands; Cardiovascular Research Institute Maastricht (CARIM), Maastricht University, Maastricht, The Netherlands

**Keywords:** periprocedural myocardial infarction, myocardial infarction, coronary artery bypass grafting, questionnaire

## Abstract

**Objectives:**

The definitions of periprocedural myocardial infarction (PMI) after coronary artery bypass grafting (CABG) are heavily debated. A European Association for Cardiothoracic Surgery (EACTS)-endorsed international survey was conducted to evaluate current clinical practices and to reach a consensus-based agreement on the diagnostic characteristics required for a potential future PMI definition.

**Methods:**

The questionnaire complied with the CHERRIES guideline. A pilot version underwent iterative testing. The final version was distributed to all members of EACTS and allied specialties. The questionnaire evaluated definitions, biomarkers, and required diagnostic accuracy and was distributed between August-November 2024.

**Results:**

The questionnaire was completed by 175 respondents (surgeons: 71.4%, cardiologists: 20.6%, intensivists: 5.1%, other: 2.9%) from 29 countries. A specific definition of PMI was used by 46.4% of respondents (Universal Definition of Myocardial Infarction [UDMI]-4: 67.4%, Society of Cardiovascular Angiography and Interventions [SCAI]: 16.3%, second Academic Research Consortium [ARC-2]: 6.1%, other: 10.2%). Respondents identified biomarker concentrations defining PMI with supporting (imaging) evidence (high-sensitivity cardiac troponin T [hs-cTnT]: 489 ng/L, high-sensitivity cardiac troponin I [hs-cTnI]: 425 ng/L, CKMB-mass: 90ug/L, CKMB-activity: 100 U/L) and without (hs-cTnT: 931 ng/L, hs-cTnI: 1458 ng/L, CKMB-mass: 100ug/L, CKMB-activity: 300UL). The lowest acceptable sensitivity and specificity thresholds for a future definition were 95% [90%-95%] and 90% [80%-95%], respectively, while sensitivity was deemed more important than specificity (79.8% vs 20.2%, *P* < .001).

**Conclusions:**

The use of PMI definitions varies widely, though UDMI-4 is most frequently employed. Our findings highlight the need for a standardized, CABG-specific PMI definition with robust diagnostic accuracy to enable consistent diagnoses and a common clinical language.

## Graphical abstract

**Figure:**
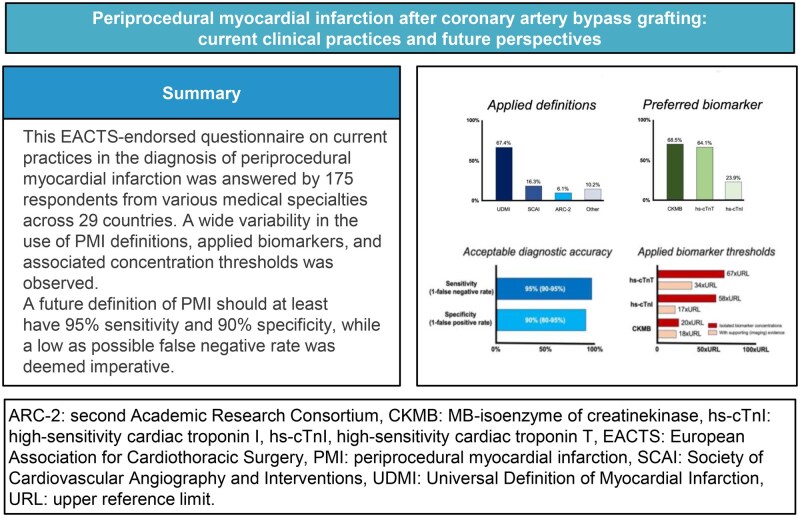

## INTRODUCTION

The diagnostic entity “periprocedural myocardial infarction” (PMI) is a widely debated topic in cardiac surgery.^1^^,^^2^ In contrast to patients with primary (or spontaneous) myocardial infarction, myocardial injury is inherent to cardiac surgical procedures in general, and to coronary artery bypass grafting (CABG) in particular.^3^ Nevertheless, it is of utmost importance to differentiate between “benign,” or inherently irreversible causes of injury, and potentially “malignant” forms of injury, such as reversible graft-related issues. Indeed, if addressed promptly, a patient’s prognosis can be ameliorated by timely intervention in case of treatable conditions.^4^ Nevertheless, a potentially life-saving intervention can only be performed when an adequate and justified diagnosis is established.

Currently, several contradictory definitions for the diagnosis of PMI have been proposed by different societies and task forces.^1^^,^^5–7^ Interestingly, the various definitions prefer different biomarkers, such as the MB isoenzyme of creatine kinase (CK-MB^6^), or cardiac troponin (cTn^5^^,^^7^). Moreover, the definitions apply different concentration thresholds of these cardiac biomarkers that may be indicative for PMI, and strongly differ regarding the requirement of ancillary criteria, such as supporting electrocardiographic (ECG) or imaging evidence. Due to these fundamental differences, the prognostic impact of these PMI definitions varies as well.^8^

Due to the differing nature in PMI definitions and their varying use, we hypothesize that there is a lack of consensus within the cardiovascular community, leaving an important knowledge gap. Therefore, the aim of this European Association for Cardiothoracic Surgery (EACTS)-endorsed multidisciplinary survey is to evaluate current practices in the diagnosis of PMI, and to reach a consensus-based agreement on the minimally required diagnostic characteristics that a definition of PMI should incorporate.

## METHODS

### Design and ethical approval

The current open and voluntary electronic survey was prepared according to the Checklist for Reporting Results of Internet E-Surveys (CHERRIES)-statement^9^ (**Supplementary Material 1** presents the checklist). As no patients were involved, and potentially eligible medical professionals were approached through official EACTS-channels, amongst others, medical ethical approval did not apply to the current study.

### Questionnaire preparation

The survey and its questions were prepared by a core group of researchers from various disciplines, such as cardio(thoracic) surgeons, (interventional) cardiologists, clinical chemistry physicians, radiologists, and intensive care physicians. After consensus was reached by the core group on the questionnaire’s content, an iterative adaptation process was initiated by testing the survey using a pilot version, which was distributed among key peers from various disciplines (the answers to these versions of the survey were not incorporated in the final analysis). The final version of the questionnaire was eventually uploaded through Surveymonkey (SurveyMonkey Inc., www.surveymonkey.com, San Mateo, CA, USA) and distributed through EACTS-channels using a Surveymonkey web-link (see below).

### Questionnaire content

In total, the survey comprised a maximum of 46 questions over 30 pages/screens, with many being optional, and an estimated time to complete of 11 min. **Supplementary Material 2** presents all the questions that were incorporated in the survey. In short, the first section was dedicated to the medical specialization and centre of the respondents. The following section consisted of questions regarding the minimally required diagnostic accuracy parameters in terms of false positive and false negative rates for a future definition of PMI. Then, we evaluated whether a specific definition for PMI was used by respondents in their centre, and which definition was preferred. The fourth section investigated which biomarkers and concentration thresholds respectively are measured, and which supporting evidence is required to determine a diagnosis of PMI. The next section determined which biomarker or combination of biomarkers are preferred to monitor the extent of periprocedural injury and investigated at what timepoint(s) biomarkers are measured postoperatively. Also, respondents were asked which indications would prompt them to perform emergent invasive coronary angiography (ICA).

For certain subjects, such as the potential use of biomarkers and their associated thresholds, 12 questioning was applied. All participants received the same questions, and we did not apply question randomization.^9^ The questionnaire was filled in anonymously, and no deidentifying or personal data were asked. Participants could only fill in the questionnaire once (no duplicate entries allowed, based on IP-addresses), and the changing of answers was not allowed.

### Participants and distribution

Participation in the questionnaire was on voluntary basis, without any (financial) incentives for participants. The questionnaire was distributed through official EACTS-channels, such as membership e-mails, LinkedIn, and X-accounts (examples are presented in **Supplementary Material 3**). In addition, various national societies were approached to stimulate the dissemination of the questionnaire to their members, such as the Dutch Society of Thoracic Surgery (NVT), the Dutch Society of Intensive Care (NVIC), the Austrian Society of Thoracic and Cardiovascular Surgery, the Portuguese Society of Cardiothoracic and Vascular Surgery, and the members of the European Society of Cardiology (ESC) working group for Cardiovascular Surgery. The survey was opened between August 2nd, 2024 and November 6th 2024, and all surveys were characterized by a timestamp, IP-address, and pseudonymized respondent identification number.

Inherent to the methods of distribution, participation rate could not be estimated, although the completeness rate per participant was calculated. Both complete and incomplete questionnaires were used for the final analysis.

### Statistical analysis

The statistics used for the presentation of the data obtained from the survey were descriptive of nature. The distribution of continuous data was tested using Kolmogorov-Smirnov’s test. In the current study’s case, all continuous variables were therefore reported as medians with corresponding interquartile ranges (25th-75th percentile). Categorical data were presented as counts and percentages (%). Of note, biomarker thresholds were reported both as absolute concentrations and normalized to their respective upper reference limit (URL). The applied URLs differed per biomarker and are presented in **Supplementary Material 4**. The association between medical specialties and application of biomarkers in clinical practice was evaluated in an unadjusted binary logistic regression model and expressed in odds ratios (ORs) with corresponding 95% confidence intervals (CIs). Finally, sensitivity analyses were performed to compare the conceptions across geographical locations based on continents towards preference for PMI definitions and required diagnostic accuracy thresholds.

These statistical analyses were performed using SPSS (version 29.0, SPSS Statistics, IBM Corp., Armonk, NY, United States).

## RESULTS

### Participants and medical specialties

**Figure 1.**
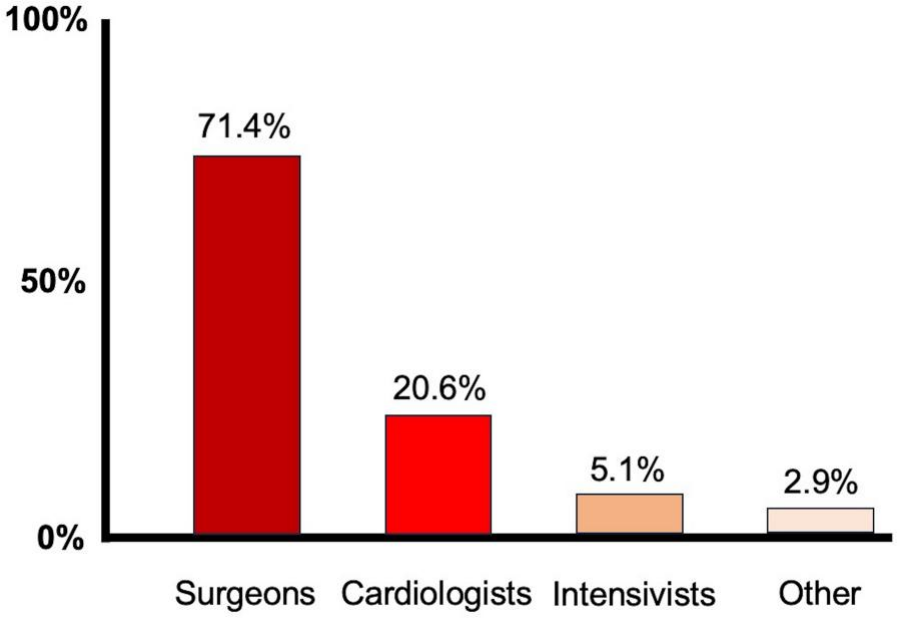
Responding Medical Specialists

Between August and November 2024, a total of 175 respondents completed the survey. Of those, 125 (71.4%) respondents were cardio(thoracic) surgeons, 7 (4.0%) were interventional cardiologists, 29 (16.6%) were non-interventional cardiologists, 9 (5.1%) were intensive care physicians, and 5 (2.9%) of respondents were physicians of other allied specialties (**Figure 1**).

### Demographics

**Figure 2.**
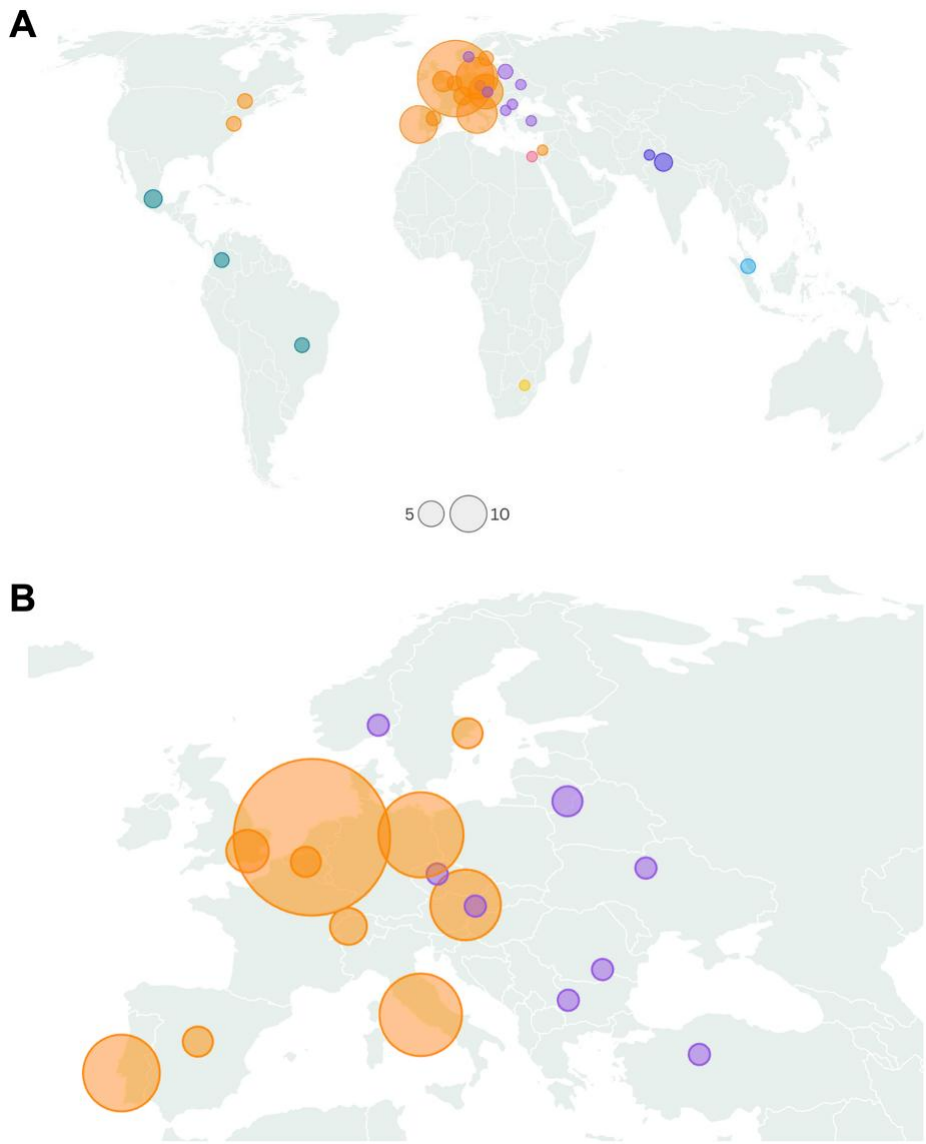
Geographical distribution of respondents on a worldwide level (A) and for Europe (B)

In total, 151 respondents (87.4%) indicated their nationality. The participants originated from 29 different countries (**Figure 2A** and **B** present a world map with a geographical distribution). Most of the respondents were from Europe. A detailed overview of participants per country is presented in **Supplementary Material 5**.

### Current use of PMI definitions

**Figure 3.**
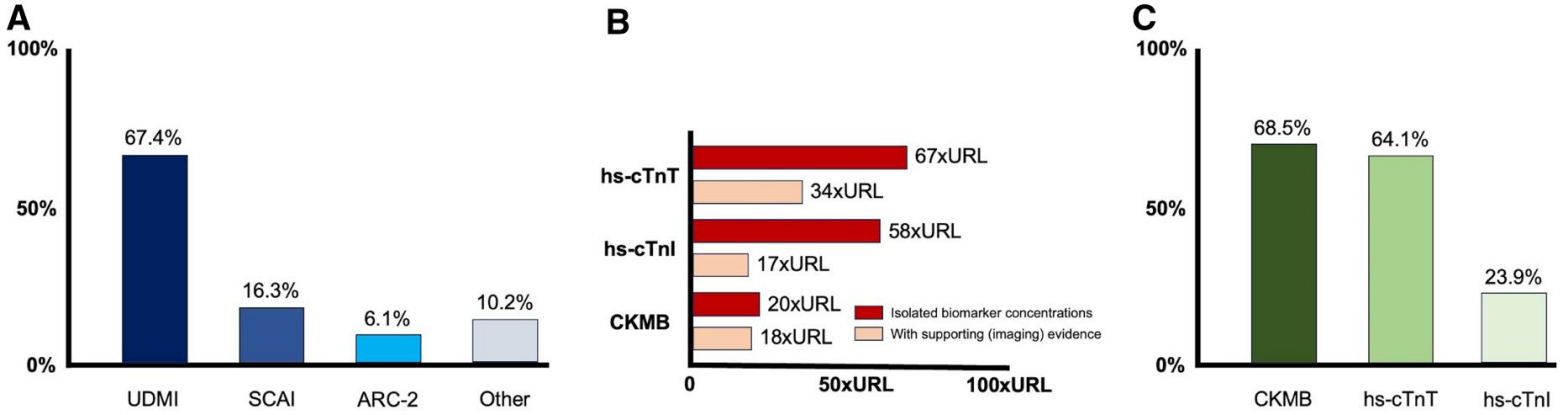
Applied definitions (A), used biomarkers (B), and associated concentration thresholds (C). Abbreviations: ARC-2: second Academic Research Consortium; CKMB: MB-isoenzyme of creatinekinase; hs-cTnI: high-sensitivity cardiac troponin I; hs-cTnT: high-sensitivity cardiac troponin T; SCAI: Society of Cardiovascular Angiography and Interventions; UDMI: Universal Definition of Myocardial Infarction; URL: upper reference limit

Less than half of the respondents (46.4%) applied a specific definition of PMI in their current practice, while 41.0% did not apply a definition, and 12.5% were not aware of PMI definitions. Of the respondents who applied a definition of PMI, 67.4% applied the Universal Definition of Myocardial Infarction [UDMI]-4,5 as compared to 16.3% for Society of Cardiovascular Angiography and Interventions [SCAI],^6^ and 6.1% for the definition as proposed by the second Academic Research Consortium (ARC-2,7 **Figure 3A**). No difference between PMI definition preference was observed among continents (*P* = .954).

### Cardiac biomarkers

Cardiac troponin was the most widely used biomarkers for the monitoring of myocardial injury (72.0% of respondents). Within these respondents, cardiac troponin T (cTnT) was measured by 77.9%, and cardiac troponin I (cTnI) was measured by 33.3% of these respondents, while most of them used the high-sensitivity assays (79.3% and 71.4% respectively for high-sensitivity cardiac troponin T [hs-cTnT] and high-sensitivity cardiac troponin I [hs-cTnI]). For hs-cTnT, there is only one vender/assay, but for hs-cTnI, only one respondent out of 20 could recall which specific vendor and assay they used.

A combination of CK and CK-MB was used by 54.6% of the respondents, while 16.2% of the respondents used CK-MB in solitude to monitor the extent of myocardial injury. The CK-MB mass assay (expressed in ng/L, 59.0%) was more frequently applied than the CK-MB activity assay (expressed in U/L, 41.0%). Other, less frequently used, biomarkers comprised aspartate aminotransferase (ASAT, *n* = 3), lactate dehydrogenase (LDH, *n* = 1), myoglobin (*n* = 4), and NT-proBNP (*n* = 2).

Interestingly, we found a significant association between medical specialty and current use of CK-MB as a biomarker. Indeed, the OR for surgeons to use CK-MB versus non-surgeons was 3.83 (95% CI 1.54-9.56, *P* = .003), while the OR for cardiologists to use CK-MB versus non-cardiologists was 0.35 (95% CI 0.14-0.91, *P* = .031). We did not observe such significant associations for the use of cTns.

Notably, when asked which biomarker was ideally preferred by the respondents (multiple answers were allowed), CK-MB was mentioned by 68.5% of respondents, while hs-cTnT (64.1%) and hs-cTnI (23.9%, together 76.1%) were most frequently stated to be preferred. In turn, CK (29.4%), NT-proBNP (8.7%), myoglobin (6.5%), and others (all <5.0%) were less frequently reported (**Figure 3B** and **3C**).

### Biomarker concentration thresholds

The applied biomarker thresholds that diagnose PMI may differ when supporting evidence such as electrocardiographic or imaging abnormalities are in play.^6^^,^^7^ For (hs-)cTnT, the threshold was 489 ng/L [140-1087] when ancillary evidence was present, compared to 931 ng/L [140-2058] without ancillary evidence. For (hs-)cTnI, this was 425 ng/L [140-2111] and 1458 ng/L [140-3585], respectively. For CK-MB (mass), these thresholds were 90 µg/L [38-100] and 100 µg/L [48-100], respectively. An overview of the biomarkers and associated concentration thresholds (both in absolute concentration and normalized to the URL) is provided in **Table 1** and **Figure 3B**.

**Table 1.** Biomarker Characteristics and Thresholds

	(hs-)cTnT (ng/L)	(hs-)cTnI (ng/L)	CK-MB-mass (µg/L)	CK-MB-activity (U/L)
Users (*n*, %)	Yes: 77.9% No: 16.9% Unknown: 5.2%	Yes: 33.3% No: 61.1% Unknown: 5.6%	Yes: 69.4% No: 31.9% Unknown: 8.7%	Yes: 42.2% No: 46.9% Unknown: 10.9%
Use of concentration threshold (*n*, %)	Yes: 31.6% No: 49.1% Unknown: 19.3%	Yes: 40.0% No: 45.0% Unknown: 15.0%	Yes: 45.3% No: 43.4% Unknown: 11.3%	Yes: 8.6% No: 67.4% Unknown: 14.0%
Ancillary evidence concentration threshold (absolute)	489 ng/L [140-1087]	425 [140-2111]	90 [38-100]	100 [37-360]
Ancillary evidence concentration threshold (URL)	34 [10-77]	17 [6-84]	18 [8-20]	4 [2-15]
Isolated concentration threshold (absolute)	931 [140-2058]]	1458 [140-3585]	100 [48-100]	300 [99-360]
Isolated concentration threshold (URL)	67 [10-147]	58 [6-143]	20 [10-20]	13 [4-15]
High-sensitivity assay	Yes: 79.3% No: 15.5% Unknown: 5.2%	Yes: 71.4% No: 14.3% Unknown: 14.3%	NA	NA

Abbreviations: CK-MB: MB-isoenzyme of creatine kinase; cTn: cardiac troponin; Hs-: high-sensitivity; NA: not applicable; URL: upper reference limit.

The majority of respondents measured biomarkers routinely after surgery (81.5%), while 13.0% did not measure routinely (but instead upon indication), and 5.4% was not aware of routine measurements. An overview of (routine) measurement timing is presented in **Supplementary Material 6**.

### Supporting evidence

ECG abnormalities that were indicative of ischaemia, prompting the need for emergent ICA, were ST-elevation without reciprocal depression (28.2%), ST-elevation with reciprocal depression (75.3%), new Q-wave formation (30.6%), left bundle branch block de novo (29.4%), and ventricular arrhythmia (ventricular tachycardia; 36.5%, ventricular fibrillation; 65.9%, **Supplementary Material 7**).

Other findings that would prompt emergent ICA were echocardiographic new regional wall motion abnormalities (77.4%), new global left ventricular dysfunction (20.2%), excessive solitary biomarker increases without other evidence (28.6%), excessive solitary biomarker increases with unspecific evidence (such as non-truly-ischaemic ECG-changes; 54.8%), and persistent haemodynamic instability (58.3%, **Supplementary Material 8**).

### Required diagnostic accuracy of an ideal definition of PMI

**Figure 4.**
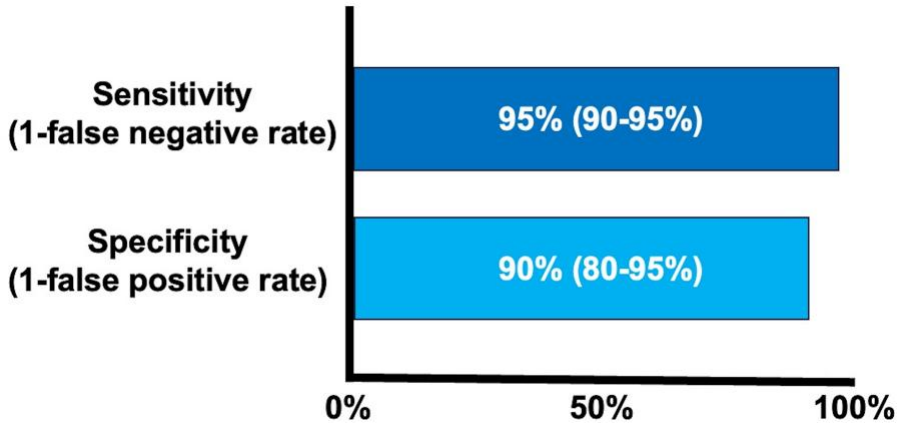
Lowest Acceptable Diagnostic Accuracy of a Potential Future Definition of PMI

When considering a potential future definition of PMI, its (minimally) required diagnostic accuracy must ideally be predetermined. Consequently, these diagnostic properties were evaluated. In our sample, the lowest acceptable sensitivity (ie, 1-false negative rate) was 95% [90%-95%], while the lowest acceptable specificity (ie, 1-false positive rate) was 90% [80%-95%]. When asked to choose between sensitivity and specificity, the majority of respondents preferred sensitivity (79.8%) over specificity (20.2%, *P* < .001, **Figure 4**). No significant differences across continents was observed towards desired diagnostic accuracy thresholds (sensitivity *P* = .142, specificity *P* = .665).

## DISCUSSION

The main findings of our study can be summarized as (i) a marked variability in the use of contemporary PMI definitions, with UDMI-4 being the most applied definition, though many respondents were not aware of the existence of PMI definitions, (ii) a marked variation in the use of cardiac biomarkers and their associated thresholds, (iii) surgeons more commonly use CK-MB as compared to non-surgeons, (iv) 68.5% of respondents preferred CK-MB as the biomarker of choice, followed by hs-cTnT (64.1%) and hs-cTnI (23.9%), and when combined, cTn was preferred by 76.1% of respondents (and therefore the most preferred biomarker), while (v) a future definition of PMI should at least have 95% sensitivity and 90% specificity, and sensitivity is deemed more important than specificity.

In cardiac surgery, several contemporary PMI definitions are applied, with their associated preferred cardiac biomarkers and different thresholds and ancillary criteria.^5–7^ Consequently, the rate of PMI in CABG populations can differ substantially depending on the applied definition.^8^ Moreover, the decision to perform additional diagnostic and therapeutic interventions (such as follow-up echocardiography or coronary angiography) following signs of ischaemia may be heavily influenced by the use of specific PMI definitions. This may in turn even affect the outcome and prognosis of the overall group of patients undergoing CABG. Although many previous studies have assessed the prognostic effect of the application of these definitions,^8^^,^^10^^,^^11^ and there is an ongoing debate to which definition is “better” than the other,^2^^,^^12^ there is an important knowledge gap in current practices and preferences, which is imperative to address before we can find a way forward as a community.

Our results indicate that more than half of clinicians apply other, perhaps less well-defined criteria to diagnose periprocedural ischaemia and PMI, since 46.4% of respondents did not use a specific definition of PMI, and 12.5% of participants were not aware of PMI definitions. Nonetheless, within the respondents who applied a definition, the majority used UDMI-4.5 The preferred use of UDMI-4 also underlines the seeming importance of supporting evidence in terms of ECG- or imaging-abnormalities when diagnosing PMI in CABG patients.

Despite the preference in 67.4% of respondents to use UDMI-4, a considerable number of participants still used, or preferred, CK-MB (in combination with CK or not) in clinical practice. This seems a cardiac surgery-specific phenomenon, in which there is conflicting evidence for the diagnostic accuracy of cardiac biomarkers. The differing results regarding the diagnostic properties of cTns and CK-MB in surgical patients may arise from non-ischaemic release mechanisms during CABG, such as the use of cardiopulmonary bypass, cardioplegic arrest, and mechanical manipulation.^3^ Previous studies have suggested that cTns are more abundantly released in this setting, and particularly cTnI, as compared to CK-MB, and cTnT.^3^^,^^13^^,^^14^ Nevertheless, the use of CK-MB, as observed in the current survey, is still supported in the latest EACTS consensus statement.^1^

In line with this consensus statement and the SCAI and ARC-2 definitions,^1^^,^^6^^,^^7^ our results suggest that respondents apply lower cardiac biomarker concentration thresholds when ancillary evidence such as ischaemic ECG changes or new regional wall motion abnormalities are present, and only diagnose PMI based on biomarker concentrations when these are exceedingly high. The suggested thresholds by our respondents to diagnose PMI independent of supporting evidence of 58-67xURL for cTns closely correspond to ARC-2 and SCAI,^6^^,^^7^ while the suggested 20xURL threshold for isolated CK-MB concentrations is actually completely in line with the EACTS consensus statement.^1^ Nevertheless, the concentration thresholds as suggested in this survey are based on expert-opinion and consensus, and not on objective data. Ideally, a future definition of PMI could be based on objectively quantified loss of viable myocardium upon cardiac imaging following surgery.

A consensus for a required diagnostic accuracy of such a future definition of PMI seemed to be reached upon a minimally required sensitivity of 95% and specificity of 90%, and respondents deemed a low false negative rate (ie, 1-sensitivity) more important than a low false positive rate (ie, 1-specificity). These diagnostic accuracy measures may be used when developing a new definition, or when determining more objective biomarker concentration thresholds that are indicative of PMI.

Finally, there are important differences in biomarker release mechanisms and abundance between surgical procedures. Our survey was specifically designed to address this knowledge gap for CABG procedures, and this may also be required for other surgical interventions such as valve procedures. This could eventually lead to procedure-specific definitions of PMI. In addition, as previously demonstrated,^15^ biomarker release may also depend on patient-specific characteristics such as renal function or procedural urgency and future PMI definition could take these features into account. Together, such efforts could facilitate the adequate application of PMI definitions across various patient populations.

### Limitations

We have attempted to give a complete overview of current practices by seeking the input from both surgeons, cardiologists, intensivists, and other allied physicians, but selection bias may apply. Also, >10% of participants stated to use an “other” definition of PMI, but these were not specifically stated. Additionally, there is a geographical imbalance that may skew the results, favouring mainly European respondents from specific countries, due to the active participation of various national societies in disseminating the survey. Although we did not observe significant differences between continents towards participants’ preference for PMI definitions and diagnostic accuracy thresholds, these analyses are likely underpowered. Therefore, our results seem particularly applicable to the (West-)European context. Furthermore, not all surveys were 100% completed, which may be the consequence of its length. In addition, our results particularly apply to on-pump CABG procedures with use of cardioplegic arrest, and results may differ for patients undergoing off-pump CABG. Therefore, our results are not necessarily generalizable to off-pump procedures or minimally invasive CABG. Finally, to reach as many participants as possible, the survey was disseminated through (official) EACTS channels, which impaired the calculation of the response rate.

## CONCLUSION

The use of PMI definitions varies widely, though the UDMI-4 is most frequently employed. CK-MB and cTns are equally used in clinical practice and are similarly preferred to quantify the extent of myocardial injury. Different biomarker thresholds are applied in the presence or absence of supporting ischaemic evidence, in line with current EACTS-recommendations. Future definitions could be enhanced through the application of clinically relevant diagnostic accuracy measures, for which sensitivity is more important than specificity.

## Supplementary Material

ezaf344_Supplementary_Data

## Data Availability

All data underlying this study is available upon reasonable request to the corresponding author.
